# Lithium Induces ER Stress and N-Glycan Modification in Galactose-Grown Jurkat Cells

**DOI:** 10.1371/journal.pone.0070410

**Published:** 2013-07-22

**Authors:** Tamás Nagy, Dorottya Frank, Emese Kátai, Rikki K. K. Yahiro, Viktor S. Poór, Gergely Montskó, Zita Zrínyi, Gábor L. Kovács, Attila Miseta

**Affiliations:** 1 Department of Laboratory Medicine, University of Pécs, Pécs, Hungary; 2 Department of Dentistry, Oral and Maxillofacial Surgery, University of Pécs, Pécs, Hungary; 3 Department of Forensic Medicine, University of Pécs, Pécs, Hungary; School of Medicine and Health Sciences, University of North Dakota, United States of America

## Abstract

We previously reported that lithium had a significant impact on Ca^2+^ regulation and induced unfolded protein response (UPR) in yeast cells grown on galactose due to inhibition of phosphoglucomutase (PGM), however the exact mechanism has not been established yet. In this study, we analysed lithium's effect in galactose-fed cells to clarify whether these ER-related changes are the result of a relative hypoglycemic state. Furthermore, we investigated whether the alterations in galactose metabolism impact protein post-translational modifications. Thus, Jurkat cells were incubated in glucose or galactose containing media with or without lithium treatment. We found that galactose-fed and lithium treated cells showed better survivability than fasting cells. We also found higher UDP-Hexose and glycogen levels in these cells compared to fasting cells. On the other hand, the UPR (X-box binding protein 1 mRNA levels) of galactose-fed and lithium treated cells was even greater than in fasting cells. We also found increased amount of proteins that contained N-linked N-acetyl-glucosamine, similar to what was reported in fasting cells by a recent study. Our results demonstrate that lithium treatment of galactose-fed cells can induce stress responses similar to hypoglycemia, however cell survival is still secured by alternative pathways. We propose that clarifying this process might be an important addition toward the better understanding of the molecular mechanisms that regulate ER-associated stress response.

## Introduction

In our diet, the major source of galactose is lactose – the main carbohydrate component of milk and dairy products while a smaller amount of galactose is also present in many fruits and vegetables [1]. Moreover, in the first six months of our lifetime lactose is the most important carbohydrate source found in breastmilk. Galactose can be transported into the cells by several glucose transporters including GLUT1, GLUT2 and insulin-independent GLUT3 transporter [2]. Galactose metabolites are also continuously produced in every cell [3] as they are integral components of glycoproteins, glycolipids and proteoglycans. External galactose is generally metabolized through glycolysis, although excess amount of it can be partially diverted to polyol pathway which could can contribute to diabetic complications [4].

Masuda et. al showed that a mood stabilizer drug, lithium was toxic for *Saccharomyces cerevisiae* when cell were grown on galactose [5]. Our earlier results also indicated that lithium significantly interfered with the galactose metabolism in *S. cerevisiae* and interestingly it also influenced calcium homeostasis [6]. Based on proteome and genome-wide transcription analyses, Bro et al. concluded that genes involved in both transcription and translation were down-regulated, whereas genes responsive to different stresses as well as genes of energy reserve metabolism (e.g. glycogen accumulation) and monosaccharide metabolism were up-regulated when *S. cerevisiae* grown on galactose were treated with lithium [7]. Although lithium is the oldest mood stabilizer which is still in use [8], its exact molecular actions are still under debate. Many excellent reviews have theorized potential mechanisms for the effects of lithium [9]. Lithium ion is a monovalent cation which has an ionic radius of 72 pm similar to the 74 pm of Mg^2+^, which puts lithium in competition for proteins which use Mg^2+^ as a cofactor. Many of these proteins are closely related to carbohydrate metabolism: inositol polyphosphate 1-phosphatase (IPPase), inositol monophosphate phosphatase (IMPase), glycogen synthase kinase-3 (GSK-3) [10] and phosphoglucomutase (PGM) are targets for lithium [5]. The latter (PGM) is a key enzyme that links galactose and glucose metabolism by catalyzing the reversible conversion of glucose-1-phosphate (Glc-1P) and glucose-6-phosphate (Glc-6P).

Lithium treatment resulted in an increased Glc-1P/Glc-6P ratio in *S. cerevisiae* which had been grown in media containing galactose as the sole carbon source [6]. Interestingly, these metabolic changes were accompanied by gross alterations in Ca^2+^ homeostasis. Vacuolar Ca^2+^ stores were considerably elevated but the basal intracellular free Ca^2+^ was normal and it's increase upon various stimuli was repressed. In the *pgm2Δ* yeast strain which lacks the major isoform of PGM, similar changes occurred when cultured in galactose containing media. Moreover, we have observed that unfolded protein response (UPR) was also greatly elevated [11]. Taken together, it appeared probable that the modulation of lithium on galactose metabolism strongly influences ER related processes such as Ca^2+^ homeostasis and UPR [6].

Disturbances in galactose metabolism may induce ER stress due to various mechanisms. First, blocking PGM by lithium might lead to a relative hypoglycemic state, since galactose can not be directly processed through glycolysis. Secondly, intracellular accumulation of galactose and intermediate metabolites such as galactose-1-P (Gal-1P) and Glc-1P can be also responsible for the ER stress [12], e.g. by increasing the amount of reactive oxygen species (ROS) [13]. During a ‘typical’ stress situation such as oxidative stress or hypoglycemia, protein malfolding precedes UPR. Proper structural folding of proteins is energy demanding [14], similarly, oligosaccharide synthesis requires carbohydrate metabolites that could otherwise be used for ATP production. Decreasing the amount or changing the structure of protein-linked oligosaccharides also serves as intermediate signal during the protein quality control. For example, Grp78 forms a more stable complex with underglycosylated proteins [15], thus UPR can be initiated and the cells will be able to save energy and attenuate (at least temporarily) the translation of unnecessary proteins. Indeed, in genetic disorders impacting galactose metabolism such as galactosemia it is thought that N-glycan assembly and processing is also defective [16]. Earlier we have found in yeast that oligosaccharide trimming and carbohydrate metabolism are strongly interacting [17], but the impact of substrate availability (or surplus) on N-glycan synthesis and UPR requires further investigation.

In this study we sought to study ER related effects of altered galactose metabolism caused by lithium treatment in mammalian cell lines. Since our previous results showed significant changes in carbohydrate metabolism, calcium regulation and ER-stress through the inhibition of PGM by lithium, our aim was to investigate whether these changes were due to a relative hypoglycemic condition. We have compared galactose-fed, lithium treated cells to starved cells, and studied the differences and similarities in survivability, metabolite levels, calcium regulation and UPR. Moreover, we found that a specific form of N-glycans recently described in starving cells [18] can also be present in galactose-grown, lithium treated cells. Our data reveal that lithium treatment in conjunction with galactose may induce intracellular defensive mechanisms characteristic for fasting condition; however, the better survivability of these cells demonstrated that alternative metabolic pathways bypassing PGM have to exist for galactose.

## Materials and Methods

### Cell line and culture conditions

Jurkat cells (ATCC TIB 152 human acute T-cell leukemia) were cultured in glucose-free RPMI 1640 medium (R1383) supplemented with 10% fetal bovine serum (FBS), penicillin (100 U/mL), streptomycin (100 µg/mL) and 5 mM of basal glucose. The cell culture was incubated at 37°C, 5% CO_2_, in a humidified incubator. Subculturing was performed every 2–3 days and fresh media was replaced 12–24 hours prior to each experiment. Cell counting was performed regularly using an Cell-Dyn 3700 hematology analyzer (Abbott) in ‘open’ mode to adjust the amount of cells and in the cell-growth experiments. At the start of each experiment cells were resuspended and subsequently incubated for up to 72 hours in fresh glucose-free media supplemented with one of the following: 1.) 5 mM glucose (glc) 2.) 5 mM galactose (gal) 3.) no carbohydrate source (Ø) 4.) 5 mM glc +2.5 mM lithium chloride (Li) 5.) 5 mM gal +2.5 mM Li 6.) Ø+2.5 mM Li 7.) 5 mM glc +10 mM Li 8.) 5 mM gal +10 mM Li 9.) Ø+10 mM Li.

Enzyme inhibition: in some of the experiments Jurkat cells were also treated with the followings: tunicamycin (Sigma-Aldrich), which inhibits the transfer of N-actelyglucosamine-1-phosphate from UDP-N-acetylglucosamine to dolichol phosphate was used at 0,5 µM, 37°C for 24 hours to prevent N-glycan synthesis. 100 µM O-(2-acetamido-2-deoxy-D-glucopyranosylidene)-amino-N-phenylcarbamate (PUGNAc; Sigma-Aldrich) was used at 37°C for 3 hours to inhibit O-GlcNAcase and O-linked N-acetylgulcosamine (O-GlcNAc) degradation.

### Apoptosis assays

Pretreated Jurkat cells were harvested and washed 2X in ice cold phosphate-buffered saline (PBS). Approximately 10^6^ cells from each group were stained for Propidium Iodide (PI) and Annexin V-FITC positivity according to the manufacturer's protocol (BD Pharmingen). The fluorescence intensity of PI dye per cell was detected at 620 nm (FL3 channel) and FITC Annexin V intensity was detected at 525 nm (FL1 channel) with Cytomics FC 500 flow cytometer (Beckman Coulter). The quadrant of live cells (negative for both PI and FITC Annexin V) was defined on control samples and identical boundaries were utilized for all samples.

### Immunoblotting

Jurkat cells pretreated as described above were washed 2X in ice-cold PBS, and harvested in a modified RIPA buffer (10 mM Tris (pH = 7.4), 100 mM NaCl, 1 mM EDTA, 1 mM EGTA, 1% Triton X-100, 10% glycerol, 0.1% SDS, 0.5% deoxycholate, protease inhibitor cocktail (1 tablet/10 mL, Roche) on ice for 30 min. and centrifuged for 10 min. at 14000 g. The protein concentration of the supernatant was measured using Bio-Rad *Dc* Protein Assay Kit (Bio-Rad). Some of the samples were subjected to PNGase F (Sigma-Aldrich) digestion, according to the manufacturers protocol. Proteins were separated on 7.5% SDS-PAGE and transferred to PVDF membrane (Millipore). Equal loading of protein was confirmed by Sypro Ruby Protein Blot Stain (Bio-Rad) prior to immunoblotting. Blots were probed either with CTD110.6, a monoclonal mouse IgM antibody (Sigma-Aldrich) (1∶2000) [19], in casein blocking buffer and followed with HRP conjugated rabbit anti-mouse IgM antibody (Pierce) (1∶5000), or HRP conjugated wheat germ agglutinin (WGA; Sigma-Aldrich) according to the manufacturer's instruction. The blots were developed using Femto chemiluminescent substrate (Pierce) and the signal was detected by Kodak Image Station 2000R (Kodak). Densitometry was quantified using Kodak 1D analysis software.

### Glycogen content

Cytospin samples were prepared and stained according to Periodic Acid Schiff (PAS) protocol [20]. Briefly, the cytospin preps were incubated in periodic acid solution for 15 minutes, followed by 20 minute incubation in Schiff's reagent. Sections were counterstained for 10 minutes using hematoxylin. At least 3000 cells were counted by light microscopy for each sample condition and glycogen content was expressed as percent of PAS positive cells.

### Ca^2+^ measurements

Intracellular free Ca^2+^ ([Ca^2+^]_i_) levels were measured by fluorescence using Fura2-AM (Life Technologies) fluorescence dye as a Ca^2+^ probe. Jurkat cells were cultured and pretreated as mentioned above. Following a quick wash in Hank's balanced salt solution (HBSS), all samples were resuspended in HBSS containing 1% bovine serum albumin, 1.2 mM Ca^2+^, 1.0 mM Mg^2+^, 2 µM Fura2-AM and the appropriate amount of glc, gal, and lithium. Samples were incubated at room temperature for 30 min. Following 2 wash steps to remove the excess of the fluorescence dye, the cells were resuspended in HBSS without Ca^2+^ but containing glucose, galactose and/or lithium as indicated above. Fluorescence signal was measured at 25°C by F4500 fluorescence spectrophotometer (Hitachi) at excitation wavelengths of 340/380 nm and emission wavelength of 510 nm. [Ca^2+^]_i_ concentrations were calculated as follows: 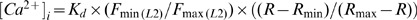. At the end of each experiment, 1.2 mM Ca^2+^, 25 µM digitonin and subsequently 10 mM EDTA were added to get maximal and minimal [Ca^2+^]_i_ signals, respectively.

Ca^2+^ stores were assessed using 2 µM magFura2-AM (Life Technologies). Cells were incubated as stated above, with slight modification. Loading occurred at 37°C and with the omission of BSA from the loading buffer to enhance the compartmentalization of the dye. Moreover, after loading, the cells were incubated at RT in loading buffer (w/o the fluorescent dye) for another 30 min. After recording the baseline fluorescence, 1 µM of ionomycin (Life Technologies) was used to elicit stored Ca^2+^ release. The ratio of the light emitted (em.: 510 nm) at excitation wavelengths of 340/380 nm was recorded and considered proportional to the Ca^2+^ concentration accessible to the magFura2 dye.

### XBP-1 expressional levels

First-strand cDNA was generated by reverse transcription of 1 µg of total RNA using High Capacity RNA-to-cDNA Kit (Life Technologies), according to the manufacturer's instructions, in a total reaction volume of 20 µL. The sequences for the reference gene were as follows: β-actin sense, 5′-AGAAAATCTGGCACCACACC-3′; antisense, 5′- GGGGTGTTGAAGGTCTCAAA-3′. The X-box binding protein 1, transcript variant 2 was amplified with these primers: sense, 5′–GCTTAGTCCGCAGCAGGT–3′; antisense, 5′-GGCTCTGGGGAAGGGCATTT-3′. Both XBP1 transcript variants were amplified with the following primers: sense, 5′-CCCAGTTGTCACCCCTCCAG-3′; the antisense primer was the same as above. Real-time PCR was performed in a LightCycler 2.0 (Roche Diagnostics) thermal cycler. Each reaction was performed in a 20 µL volume, using the ABsolute QPCR Capillary Mix (Thermo Scientific). Dissociation curves were generated after each quantitative PCR run to ensure that a single specific product was amplified. Both target and reference genes were amplified with efficiencies near 100% and within 5% of each other. For the relative gene expression analysis, the 2ΔΔCt (Livak) method was used. The expression level of the gene of interest was compared with the level of β-actin in each sample. These relative expression rates were then compared between the treated and untreated samples.

### Nucleotide sugars

Approximately 10^6^ Jurkat cells were taken from each of the pretreated samples and washed in ice-cold PBS. After centrifugation at 10000 g for 10 sec, the pellets were precipitated with icecold 0.3 M perchloric acid (PCA). PCA was extraced from the supernatant with 2 volumes of 1∶4 trioctylamine:freon mixture. HPLC was carried out as described earlier [21]. Briefly, samples were loaded on an anion exchange HPLC column (Partisil 10 SAX, Beckman) and nucleotide sugars were measured at 262 nm using 2 mL/min flow rate and linear salt and pH gradient from 5 to 750 mM (NH_4_)H_2_PO_4_ and from pH 2.8 to 3.7 respectively.

### Data analysis

Data are presented as means ± standard deviations (SD) throughout. Comparisons were performed using Students T-test and statistically significant differences between groups were defined as *P* values <0.05 and are indicated in the legends of figures.

## Results

### Growth properties of Jurkat cells

Since PGM is inhibited by lithium, galactose fed cells may experience fasting condition if they can not circumvent this blockage. Therefore, in each experiment we compared galactose grown, lithium treated cells with cells that had been carbohydrate-starved. Thus, Jurkat cells were kept in media containing either 5 mM glucose or 5 mM galactose or hexose-free media for 48 hours, while each group was also further divided into various subgroups according to the concentration of lithium (0, 2.5 or 10 mM). The number of cells was counted at the beginning of the experiment and after the first and second day.

**Figure 1:**
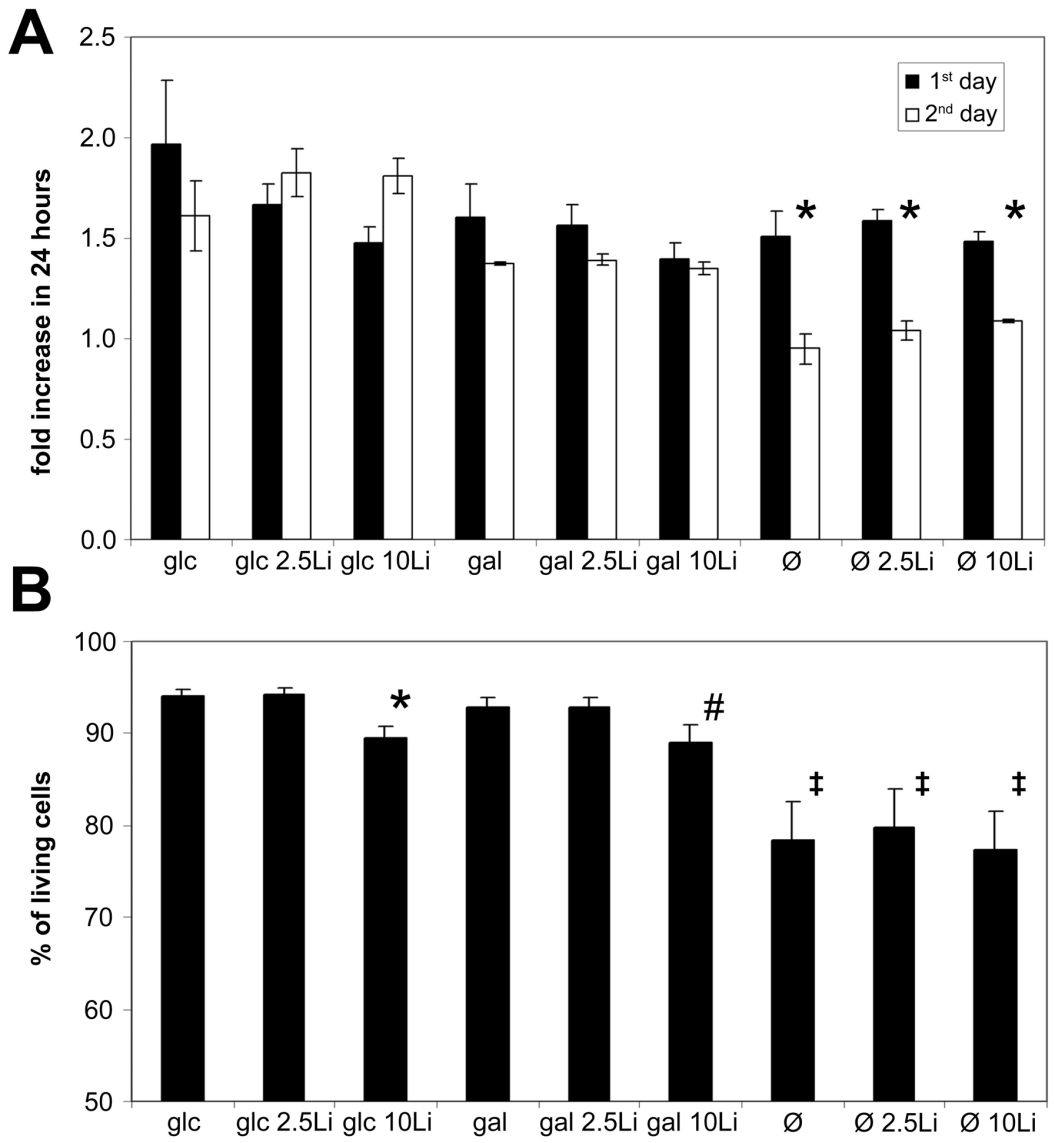
Growth potential of lithium treated Jurkat cells grown on galactose differ from fasting cells. Jurkat cells were incubated in 5 mM glucose or 5 mM galactose containing or hexose-free media for 48 hours. Lithium concentrations were adjusted for each condition at 0, 2.5 or 10 mM. (**A**) Cell counting was performed using an Abbott Cell-Dyn 3700 Hematology Analyzer at 0, 24 and 48 hours of the incubation. Cell growth is expressed as a fold increase during 24 hour periods, measured at day 1 and 2. Data are means ±SD from at least 4 independent experiments. *P<0.05 vs. 24 hours earlier. (**B**) Jurkat cells were incubated as described above, after 48 hours the ratio of living cells (Annexin V and propidium-iodide negative) were measured using flow cytometry. Data are means ±SD from at least 4 independent experiments. *P = 0.056 vs. control (glc), ^#^P<0.05 vs control (glc), ^‡^P<0.01 vs control (glc).

Cells grown in glucose doubled their numbers by the end of first day and as expected, grow somewhat more slowly in the next 24 hours (Figure 1A). Cells grow ∼20% slower in the presence of galactose compared to glucose. It has to be also noted that Jurkat cells utilize external galactose but only if no glucose is present in the media (Figure S1A). There was no significant difference between the growth rates of cells grown in the presence or absence of lithium.

Utilizing their reserves, starving cells grow similarly to cells grown in glucose or galactose containing media during the first day, but run out of fuel and grow significantly slower during the next day.

Figure 1B shows that the viability of lithium treated cells in the glucose, galactose or hexose free complemented media were similar to those measured in lithium free media. As expected, starving cells had a significantly higher probability of death compared to glucose or galactose grown cells. The addition of 10 mM lithium in both glucose and galactose fed groups seemed to only modestly decrease (∼5%) the number of living cells.

### Nucleotide sugar levels

**Figure 2:**
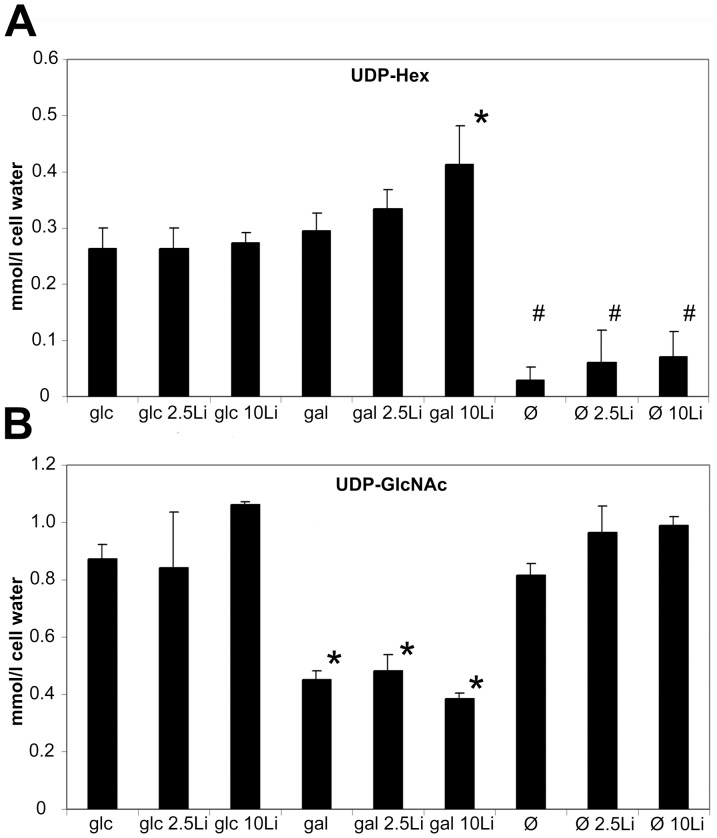
Lithium elevates UDP-Hexose content in galactose-grown Jurkat cells. (**A**) UDP-Hexose (UDP-Hex) [sum of UDP-Glucose (UDP-Glc) and UDP-Galactose (UDP-Gal)] levels from 5 mM glucose, 5 mM galactose treated or fasting cells, with 0, 2.5 or 10 mM Li treatment. After 48 hours incubation, UDP-Hex levels were measured using HPLC and expressed as mmol/cell water. Data are means ±SD from at least 4 independent experiments. *P<0.05 vs. control (glc), ^#^P<0.01 vs control (glc). (**B**) UDP-N-acetylglucosamine (UDP-GlcNAc) levels from samples pretreated as described above. UDP-GlcNAc levels were measured using HPLC and expressed as mmol/cell water. Data are means ±SD from at least 4 independent experiments. *P<0.01 vs. control (glc).

Jurkat cells were treated for 48 hours with 0, 2.5 or 10 mM lithium in 5 mM glucose, 5 mM galactose or hexose free media. In order to elucidate whether lithium treatment affected UDP-sugar metabolism we measured UDP-Hexose (UDP-Hex;  = UDP-Galactose +UDP-Glucose) levels. HPLC results revealed that glucose and galactose grown cells had similar UDP-Hex levels, while lithium significantly increased UDP-Hex, but only in galactose grown cells (Figure 2A). Glucose fed cells demonstrated very stable UDP-Hex levels in the absence or presence of various amounts of lithium. Galactose fed cells had similar UDP-Hex levels compared to glucose grown cells while the addition of lithium here increased the UDP-Hex level in a dose dependent fashion. Fasting depleted all UDP-Hex from the cells, indicating that the cells used up all their carbohydrate resources.

Somewhat unexpected, we detected decreased level of UDP-N-acetyl-glucosamine (UDP-GlcNAc) in all galactose-grown samples. UDP-GlcNAc remained relatively unchanged in starved cells (Figure 2B). Earlier, similar changes has been observed in galactosemia, namely that excess amount of galactose can indeed decrease UDP-GlcNAc levels [22]. It is likely that the elevated level of UDP-galactose (UDP-sink) is responsible for the removal of UTP stores, or that Galactose-1P inhibits UDP-GlcNAc pyrophosphorylase.

**Figure 3:**
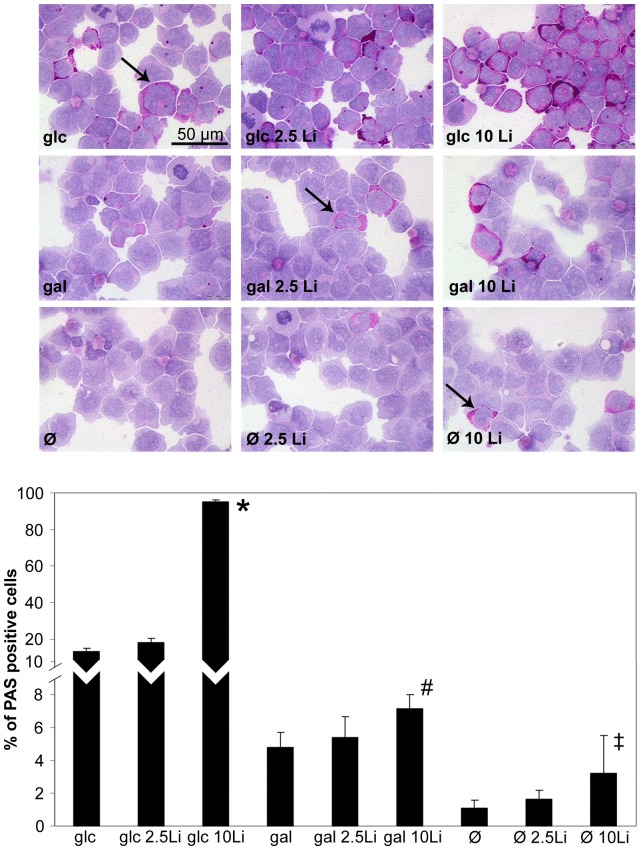
Lithium increases the glycogen content of glucose or galactose-grown and fasting cells. Jurkat cells were incubated in 5 mM glucose or 5 mM galactose containing or hexose-free media for 48 hours. Lithium concentration was adjusted for each condition at 0, 2.5 or 10 mM. After incubation, cytospin preps were stained with PAS to visualize glycogen content. Above: Representative images for each condition. PAS positivity was considered when the cell exhibited strong violet coloration in the cytoplasm (indicated by the arrows). Below: Percent of PAS positive cells. Data are means ±SD from at least 3 independent experiments, each bar represents the average of at least 3000 counted cells. *P<0.01 vs. glc, ^#^P<0.01 vs gal, ^‡^P<0.05 vs Ø.

Next, we measured the glycogen content of Jurkat cells (Figure 3). It is recognized that PAS staining positivity is common among acute leukemias [23]. Indeed, we had strong PAS positivity in control Jurkat cells. Galactose fed cells had somewhat lower glycogen levels while starved cells had hardly any remaining PAS positivity. Lithium caused an increase in glycogen content under all of the tested conditions. Glucose-fed cells accumulated the highest amount of glycogen in response to lithium treatment, but galactose-fed and starving cells also showed a dose-dependent increase of PAS positive glycogen stores upon lithium treatment.

### Ca^2+^ homeostasis

**Figure 4:**
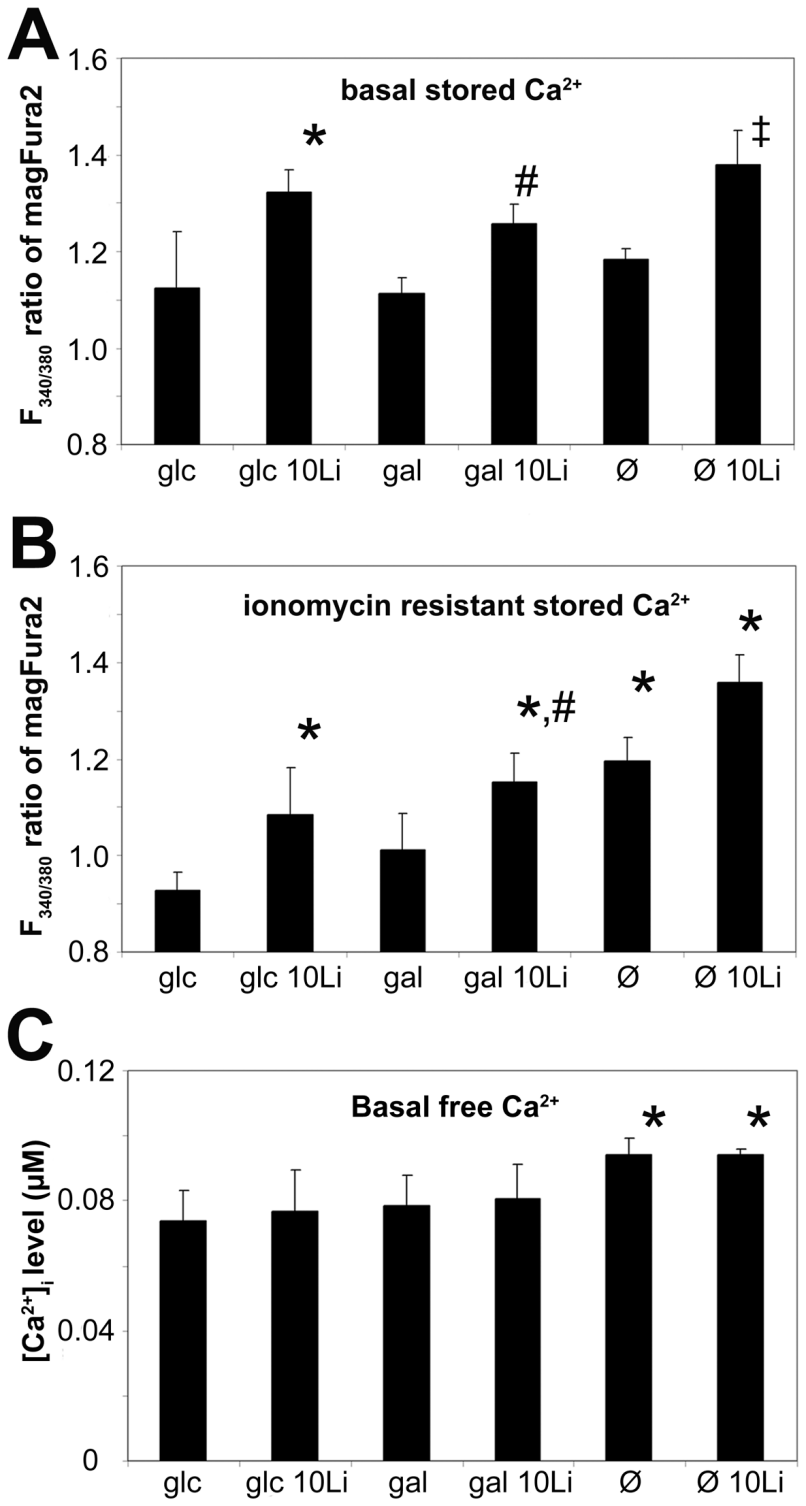
Lithium elevates stored Ca^2+^ but does not directly influence basal free Ca^2+^ levels. Jurkat cells were incubated in 5 mM glucose or 5 mM galactose containing or hexose-free media for 48 hours, with or without 10 mM lithium. (**A**) Basal ER Ca^2+^ levels were measured as the ratio of F340/F380 nm fluorescence of magFura2-AM, in the absence of extracellular Ca^2+^. *P<0.01 vs. glc, ^#^P<0.01 vs gal, ^‡^P<0.05 vs Ø. (**B**) After recording the initial stored Ca^2+^ levels, 1 µM ionomycin was used to deplete Ca^2+^ stores. The bars represent the remaining Ca^2+^ levels after steady-state is reached as a fluorescence ratio of magFura2-AM. *P<0.01 vs. glc, ^#^P<0.05 vs gal, ^‡^P<0.05 vs Ø. (**C**) Average basal intracellular free Ca^2+^ levels ([Ca^2+^]_i_) of Jurkat cells. Bars represent the calculated baseline [Ca^2+^]_i_ concentration of Fura2-AM loaded cells in the absence of extracellular Ca^2+^. *P<0.01 vs. glc. Data are means ±SD from at least 4 independent experiments.

We have previously seen that stored Ca^2+^ levels can be increased by lithium in yeast [6]. In the current study we were able to ascertain that baseline ER Ca^2+^ levels (Figure 4A) were elevated by lithium. The ionomycin resistant Ca^2+^ pool (Figure 4B) was also influenced by lithium in all cases. In lithium treated and not treated cells as well, galactose nutrition slightly, starvation strongly increased the ionomycin resistant Ca^2+^ pool compared to control.

We also measured the basal level of free Ca^2+^ in the cytoplasm by Fura2-AM dye (Figure 4C). Here we found that lithium had no significant modulatory effect on basal intracellular free Ca^2+^ ([Ca^2+^]_i_) levels in neither the glucose nor galactose grown cells nor in the fasting cells. However, fasting cells had the highest [Ca^2+^]_i_ levels.

### Unfolded protein response

**Figure 5:**
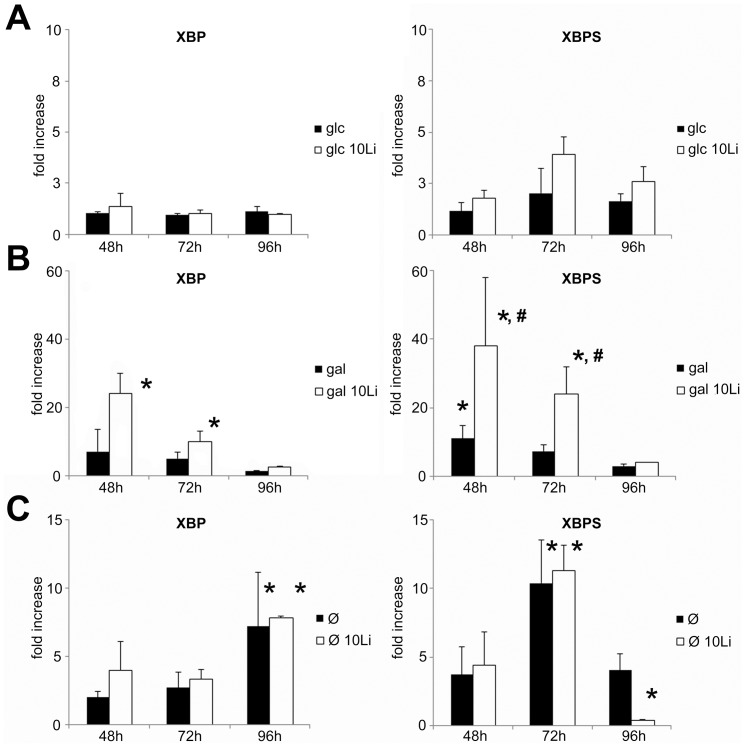
UPR is greatly activated in Jurkat cells grown on galactose and treated with lithium. Jurkat cells were incubated in 5 mM glucose or 5 mM galactose containing or hexose-free media for 48, 72 or 96 hours, with or without 10 mM lithium. UPR activation was assessed by the increase of X-box binding protein 1 (XBP) and XBP transcript variant 2 (XBPS) mRNA expressional level. Each bar represent fold increase of relative levels of XBP or XBPS mRNA compared to control (5 mM glc treated for 48 hours) samples. (**A**) Relative levels of XBP and XBPS mRNA in glucose or glucose + lithium treated cells at the indicated treatment times. (**B**) Relative levels of XBP and XBPS mRNA in galactose or galactose + lithium treated cells. (**C**) Relative levels of XBP and XBPS mRNA in fasting or fasting + lithium treated cells. Data are means ±SD from at least 4 independent experiments. *P<0.05 vs. glucose treated cells at the corresponding treatment times, ^#^P<0.05 vs. gal at the corresponding treatment times.

Earlier we showed that *pgm2Δ* mutant *S. cerevisiae* that lacks 90% of PGM activity had an extremely increased UPR when grown in galactose [11]. We have also shown that the addition of lithium to galactose grown *S. cerevisiae* inhibits PGM [6] and we recently found that it activates UPR in a similar fashion (unpublished data). Here, we used Jurkat cells grown in glucose containing, galactose containing or hexose-free media and treated with or without lithium for 48, 72 or 96 hours. Subsequently, mRNA level of XBP1 and its splicing variant were measured by RT-PCR (Figure 5).

Compared to control, XBP and XBPS levels significantly increased when cells were treated with lithium and galactose, while fasting cells did not show significantly elevated XBP or XBPS expression levels at 48 hours. Lithium had no significant effect on Jurkat cells kept in glucose containing media, or on fasting cells. However it amplified the UPR activation in galactose grown cells: XBPS were significantly elevated compared to galactose-only treated cells. After longer treatment times (72 or 96 hours), both XBP and XBPS expressional levels gradually decreased in lithium treated, galactose-grown cells compared to cells treated for just 48 hours. On the other hand, fasting cells increased their XBP and XBS expressional levels later than galactose/lithium treated cells. XBP reached its peak at 96 hours, while XBPS at 72 hours in fasting cells. Interestingly, XBPS switched back to lower levels at 96 hours.

### Protein post-translational modification

**Figure 6:**
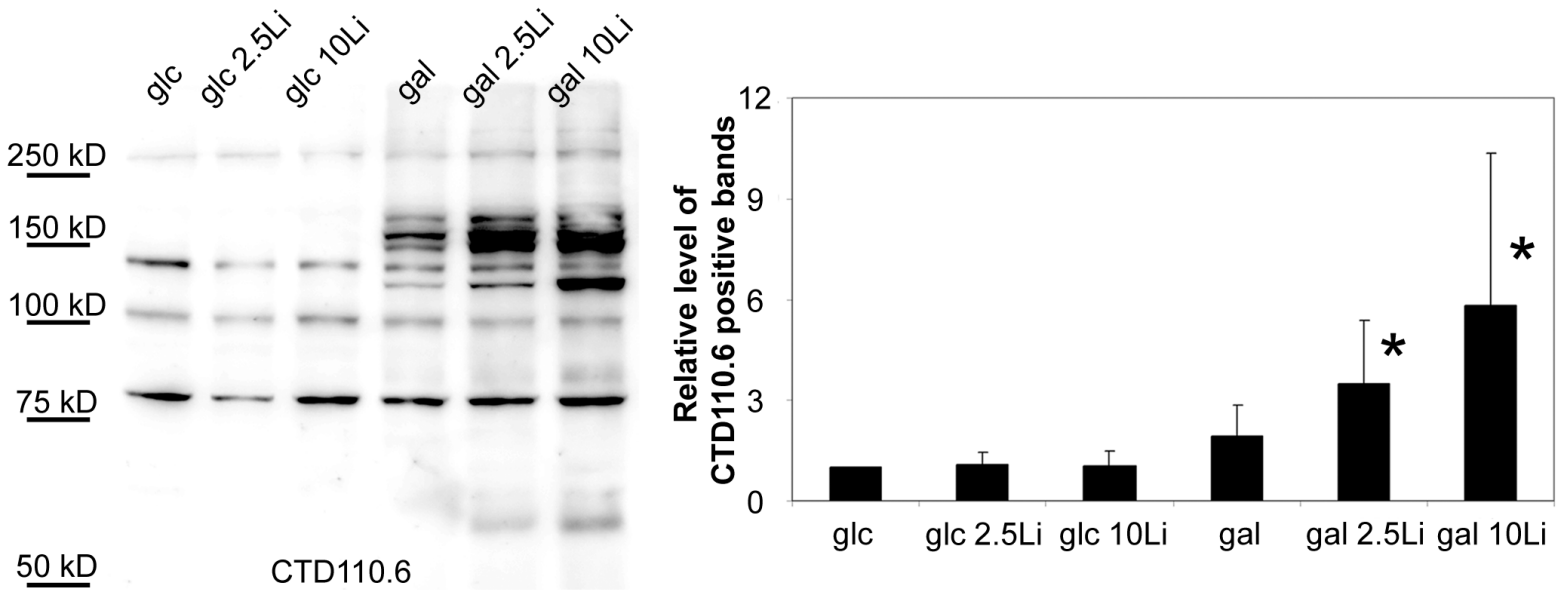
Lithium treated, galactose-fed cells show dose-dependent, apparent increase of O-GlcNAc proteins. Immunoblotting with CTD110.6 antibody in protein extracts from Jurkat cells. Cells were incubated in 5 mM glucose or 5 mM galactose containing media for 48 hours, with 0, 2.5 or 10 mM lithium. Left: Representative western-blot analysis showing cellular extracts of Jurkat cells separated using SDS-PAGE and labelled with CTD110.6 antibody. Right: Densitometric analysis of CTD110.6 positive bands. Relative levels are expressed as fold increase compared to the control, 5 mM glucose-grown cells. Data are means ±SD from 4 independent experiments after normalized for total protein staining. *P<0.05 vs. glc.

We have previously found that Jurkat cell extracts exhibit O-linked N-acetylglucosamine (O-GlcNAc) positivity when detected by CTD110.6, an anti-O-GlcNAc antibody [24]. Galactose treatment alone did not significantly increase the amount of positive proteins in Jurkat cells (Figure 6 and Figure S1B). Lithium - dose-dependently - significantly augmented the effect of galactose, but glucose grown cells did not show any significant changes upon lithium treatment.

**Figure 7:**
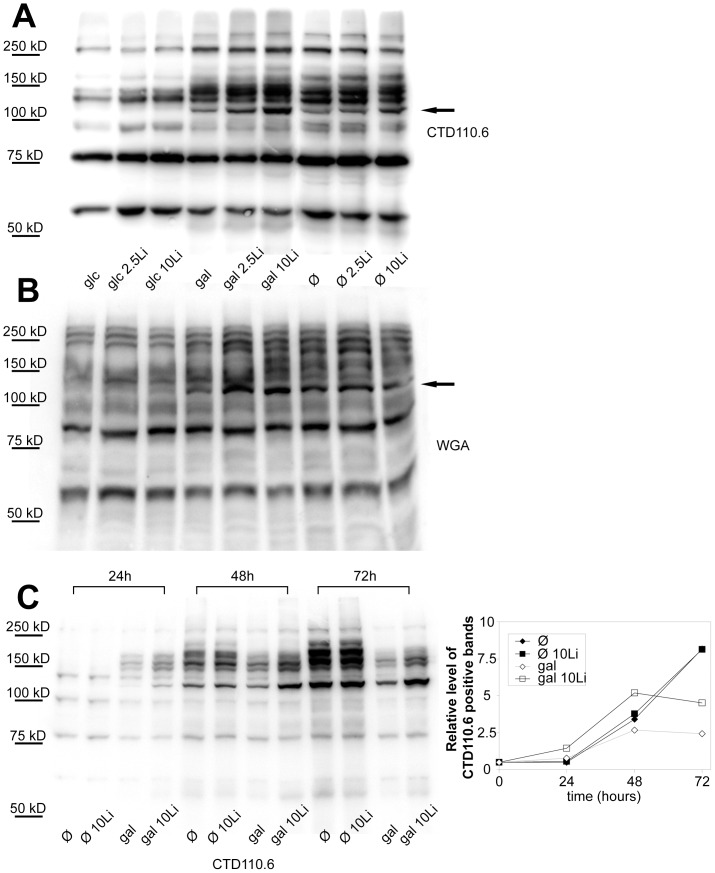
The elevation of O-GlcNAc (CTD110.6-positive) proteins in lithium treated, galactose-grown cells is similar to those of fasting cells. (**A**) Western-blot analysis using CTD110.6 antibody and (**B**) peroxidase-conjugated lectin (WGA) staining (bottom) shows representative samples of protein extracts from Jurkat cells previously incubated for 48 hours in 5 mM glucose, 5 mM galactose or hexose-free media, supplemented with either 0, 2.5 or 10 mM lithium. (**C**) Left: Representative western-blot analysis using CTD110.6 antibody (top) shows Jurkat samples previously starved or incubated in 5 mM galactose containing media, with or without 10 mM lithium. Cellular extracts were isolated on the 1^st^, 2^nd^ and 3^rd^ day of the experiment. Right: Densitometric analysis of the total CTD110.6 staining over time. Levels are expressed as a percentage of the baseline intensity. Each data point represents the average of at least 3 separate experiments. In the same order as the samples are presented on the blot from left to right, the numerical mean±SD values are: 0,71(±0,05); 0,79(±0,08); 1,06(±0,03); 1,74(±0,03); 3,59(±0,40); 4,02(±0.47); 2,83(±0.14); 4,95(±0.29); 7,88(±1.44); 7,95(±1.50); 2,26(±0.30); 3,96(±1.26), respectively.

**Figure 8:**
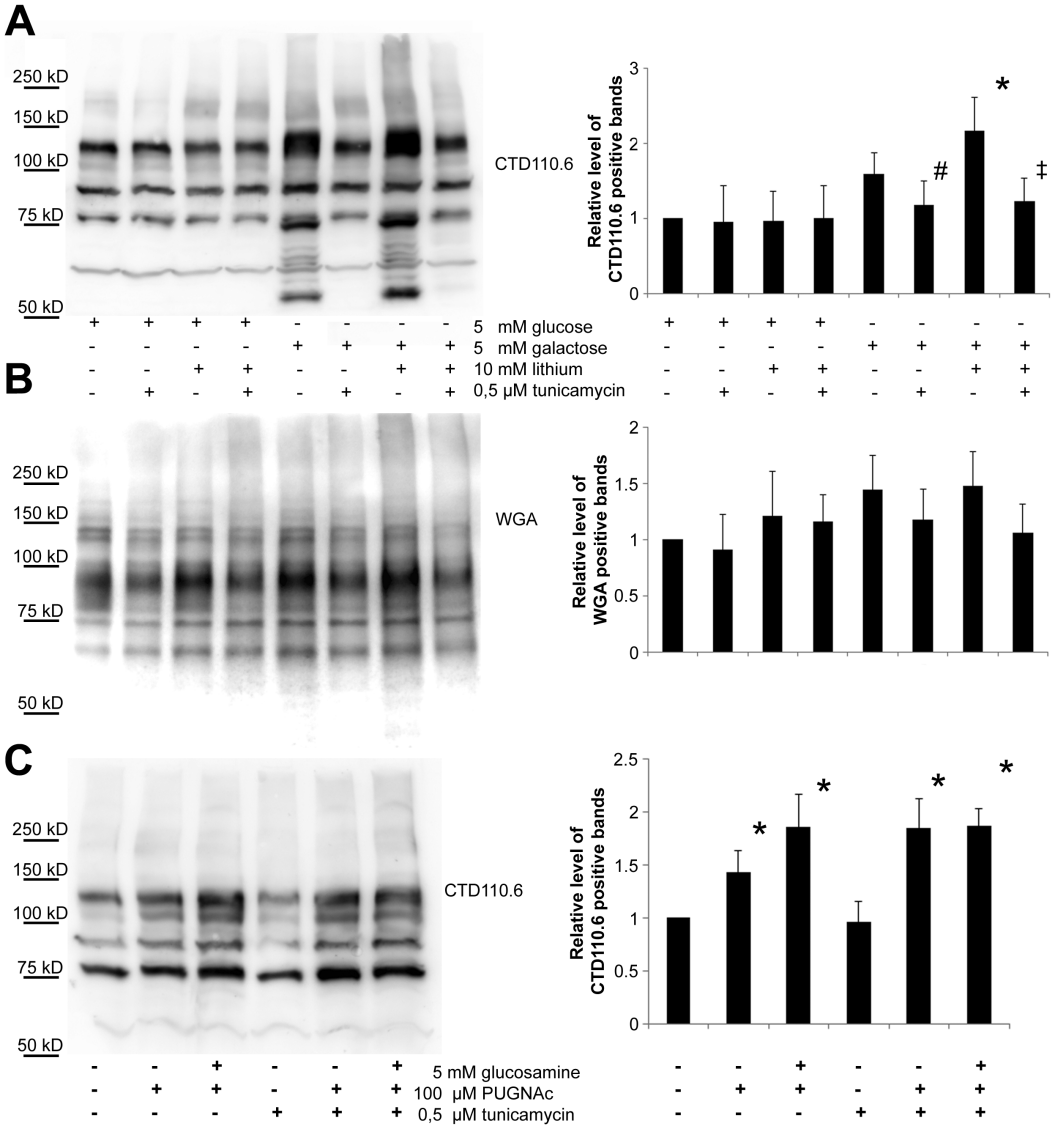
The elevated level of CTD110.6 positive proteins induced by lithium and galactose treatment can be abolished by blocking N-glycan synthesis. Immunoblot analysis of Jurkat cellular extracts stained with CTD110.6 antibody or peroxidase-conjugated lectin (WGA). Cells were grown in 5 mM glucose or galactose containing medium, with or without 10 mM lithium for 48 hours. Each group was also treated with or without 0,5 µM tunicamycin for the second 24 hours of the experiment. (**A**) Western blot analysis showing cellular extracts (30 µg/lane) separated using SDS-PAGE and stained with CTD110.6. (**B**) The same extracts were loaded on a separate SDS-PAGE and stained by peroxidase-conjugated WGA. (**C**) Western blot analysis showing protein extracts from cells kept in 5 mM glucose containing media and treated with either 5 mM glucosamine, 100 µM PUGNAc or both for 3 hours to increase O-GlcNAc levels. Starting 24 hours earlier and during the experiment 0,5 µM tunicamycin treatment was employed to exclude any N-glycan related changes. Right: Densitometric analysis of CTD110.6 or WGA positive bands. Relative levels are expressed as fold increase compared to the control, 5 mM glucose-grown cells. Data are means ±SD from 3 independent experiments after normalized for total protein staining. *P<0.05 vs. 5 mM glc, *^#^P*<0.05 vs. 5 mM gal, *^‡^P*<0.05 vs. 5 mM gal/10 mM Li.

Since blocking PGM by lithium if galactose is the only carbon source could reproduce hypoglycemia, we compared the O-GlcNAc content of starved cells and galactose fed cells (Figure 7 and Figure S2). We have found that the CTD110.6 antibody labelled proteins showed a similar increase in starved cells and in lithium treated, galactose grown cells (Figure 8A). Although less prominently, but WGA staining which binds to any glycans ending with either GlcNAc or sialic acid also showed an increase in both starved and galactose/lithium fed cells (Figure 7B).

While the staining patterns of starved and lithium-treated, galactose grown cells were very similar, starving cells showed great fluctuations in CTD110.6 intensities (Figure S2). Thus, we followed up the amount of CTD110.6 positive proteins over time (Figure 7C). We found that the amount and intensity of the CTD110.6 positive bands in starving cells can elevate to a very high level but it need some time to develop. On the other hand, the level of CTD110.6 positive proteins elevated earlier in galactose/lithium treated cells, but stabilized about 48 hours after the start of the experiment.

It was shown recently that CTD110.6 antibody might cross-react with N-linked GlcNAc_2_ containing proteins if cells are exposed to hypoglycemia [18]. To clarify whether the elevation of CTD110.6 positive bands observed in galactose grown and lithium treated cells could be also attributed to an increase of N-linked GlcNAc_2_ instead of O-GlcNAc, we treated these cells with tunicamycin, which blocks N-glycan synthesis, but O-GlcNAc is not effected by it directly. As shown in Figure 8A, tunicamycin did not alter the CTD110.6 positivity in control (glucose-fed) cells. However, it completely prevented the build-up of CTD110.6 positive proteins in both galactose-grown and galactose-grown plus lithium treated cells. Staining the same samples with WGA showed that each group treated with tunicamycin had lower amount of WGA positive proteins (Figure 8B).

**Figure 9:**
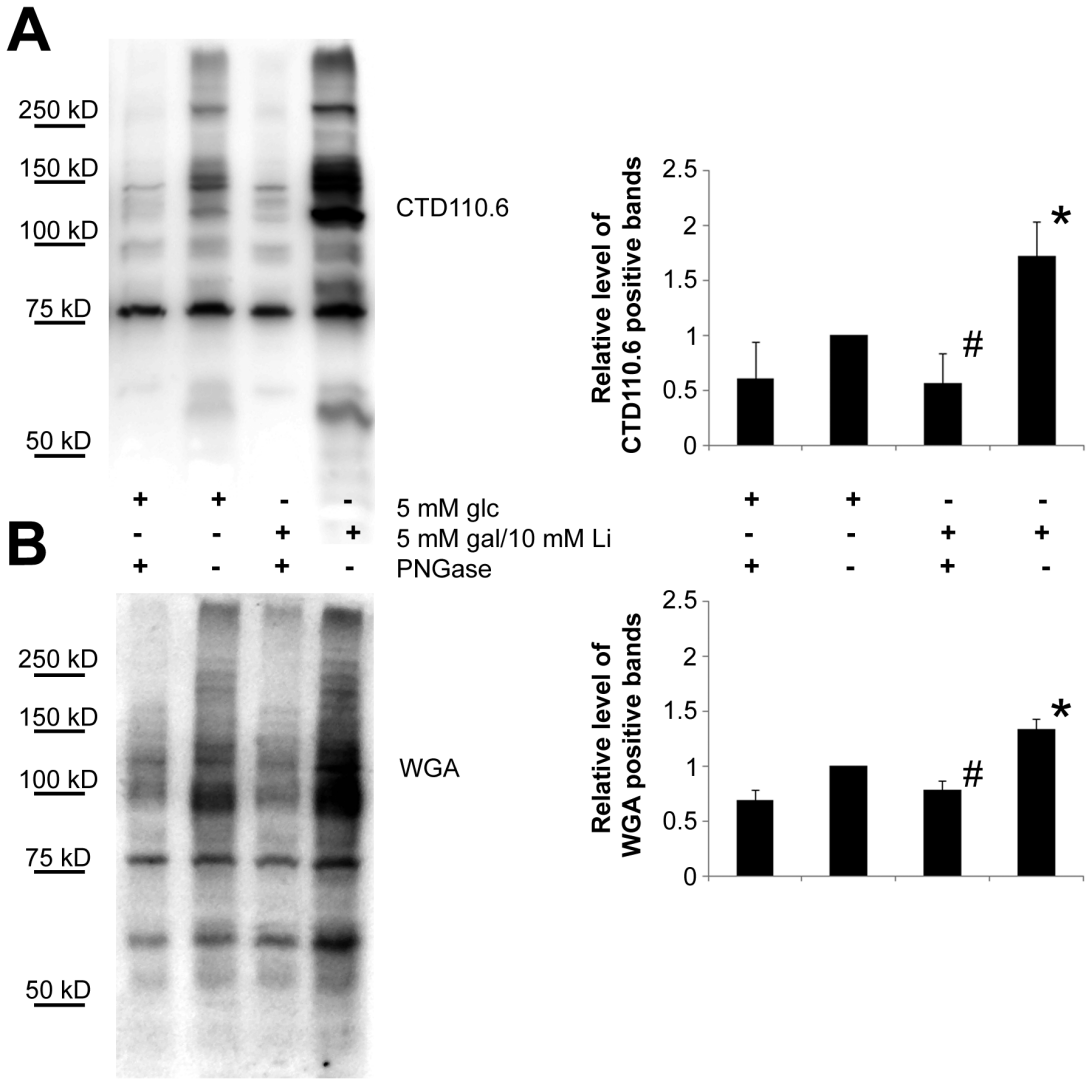
Enzymatically removing N-linked oligosaccharides disrupts elevated CTD110.6 signal in lithium treated, galactose-grown Jurkat cells. Western-blot analysis showing that CTD 110.6 antibody detects proteins with O-linked GlcNAc and Asn-linked GlcNAc. Western blot analysis showing protein extracts from cells kept in 5 mM glucose or 5 mM galactose and 10 mM lithium containing media for 48 hours. To remove all N-glycan moieties from the extracted proteins, each samples were also subjected to PNGase F digestion before loading. Following SDS-PAGE, PNGAse treated and non-treated samples were stained with either CTD110.6 antibody (**A**) or WGA (**B**). Right: Densitometric analysis of CTD110.6 or WGA positive bands. Relative levels are expressed as fold increase compared to the control, 5 mM glucose-grown cells. Data are means ±SD from 3 independent experiments after normalized for total protein staining. *P<0.05 vs. 5 mM glc, *^#^P*<0.05 vs. 5 mM gal/10 mM Li.

We could completely revert the elevated levels of CTD110.6 positive proteins in galactose/lithium treated cells by tunicamycin, but when we specifically induced O-GlcNAc modification by either glucosamine or PUGNAc which prevents the removal of O-linked GlcNAc from proteins, tunicamycin was insufficient to block the effect of glucosamine or PUGNAc (Figure 8C). Thus, in Jurkat cells the appearance of CTD110.6 positive proteins seemed to be more related to N-glycan synthesis than to O-GlcNAc modification. However, it was still plausible that the enhancement of CTD110.6 positivity we saw in lithium treated, galactose grown cells was the sign of a genuine O-GlcNAc elevation that was triggered after ER stress and UPR. Therefore we subjected protein extracts from control (glucose-fed) and galactose-fed, lithium treated cells to PNGase F enzymatic digestion that removes all Asn-linked oligosaccharide moiety but leaves Ser/Thr linked O-GlcNAc intact. As shown in Figure 9A, PNGase treatment reduced the CTD110.6 positivity of galactose/lithium treated cells nearly to the same level as control cells. WGA staining of the same samples revealed that PNGase treatment indeed removed the majority of oligosaccharides from the protein extracts (Figure 9B).

Summing up our immunoblot data, the robust CTD110.6 increase in galactose-fed, lithium treated cells proved to be similar in nature to fasting cells, moreover it was related to changes of N-glycan synthesis rather than O-GlcNAc modification. Since CTD110.6 labelling can be blocked competitively by excess amount of N-acetyl-glucosamine (data not shown), we suggest that these proteins are rich in short, GlcNAc-ending N-glycans, most probably similar or identical to the GlcNAc_2_ moiety described by the recent work of Isono [18].

## Discussion

It has been previously demonstrated that inhibiting PGM by lithium in yeast cells impacted cell growth, intracellular Ca^2+^ regulation and induced UPR, but only if the cells were grown on galactose [5], [6], [11]. We also reported that a mutant strain of yeast lacking glucose-phosphorylating enzymes when grown on galactose displayed altered Ca^2+^ homeostasis and accumulated excessive amount of glucose in the ER due to the core oligosaccharide trimming mechanism [17]. In the present study we demonstrated that altered galactose metabolism influences ER-related processes like UPR and oligosaccharide synthesis in human (T-cell derived Jurkat) cells. Moreover we found abundant amount of glycoproteins that carried altered oligosaccharide moiety, most likely GlcNAc_2_ Finally we found that the intracellular response to PGM inhibition in galactose grown-cells was similar to the stress response of starved cells (elevation of UPR, GlcNAc_2_) while the cells (in contrast to starved cells) still retained some of their growth capacity. These result suggest that lithium can modulate protein post-translational modification by influencing galactose metabolism. These findings may have important implications for the understanding of several diseases where N-glycan regulation is affected; e.g. galactosemia, autoimmun diseases or central nervous system (CNS) disorders such as Alzheimer's disease (AD) or bipolar disorder.

Proliferation of cell cultures maintained in media containing solely galactose as the carbohydrate fuel is possible but not without consequences [25]. We found that Jurkat cells kept in glucose and galactose containing media switched to galactose consumption as soon as the media ran out of glucose, at the same time lactate accumulation in the media stopped but remained stable (data not shown). Meanwhile the cells continued to proliferate at a steady, albeit slower pace (Figure S1). Since lithium inhibits PGM [6] and external galactose might not reach glycolysis, it is reasonable to assume that lithium treated, galactose-grown cell cultures would show characteristic signs of starvation: decline of the proliferation, increased rate of apoptosis, cell cycle arrest. Surprisingly, despite cellular stress was apparently present when galactose grown cells were treated with lithium, it did not cause irreversible changes such as apoptosis or the arrest of the cell cycle well known in starved cells. However, we found that cells kept in galactose - even in the presence of lithium - continued to grow. This could be explained by bypass routes that are available for the cells to utilize galactose. E.g. the Leloir pathway - including the enzymatic activity of galactose-1-phosphate-uridyltransferase (GALT) and UDP-galactose-4′-epimerase (GALE)- can convert galactose to UDP-Glc. Lithium is known to inhibit GSK-3 [10] thereby stimulates glycogen synthesis from UDP-Glc. Our present findings, namely increased UDP-Hex and glycogen levels in lithium treated, galactose grown cells suggest that at least some of the galactose entering the cell is diverted to this path. Galactonate and its downstream metabolites might also serve as an alternative pathway for galactose that circumvents glycolysis. We also can not exclude the possibility that PGM blockage by lithium was not complete. Lithium is taken up by the cells through sodium channels [26], thus 10 mM extracellular lithium probably produces intracellular lithium concentration that far exceeds human PGM IC_50_ of 1.5 mM (at 1 mM Mg^2+^ concentration) [5], however it is still possible that PGM was not inhibited completely.

Ca^2+^ regulation is altered in hypoglycemic conditions; deprivation of glucose has been reported to increase the free cytosolic [27], decrease the releasable pool [28] and increase the total ER Ca^2+^ accumulation [29]. Parallel to this findings we showed earlier that PGM inhibition by knock out models or by lithium in yeast resulted in significant Ca^2+^ accumulation and paradoxically reduced Ca^2+^ response to acut stimuli [6]. Others have also shown that in galactosemic cells ER stress was accompanied with altered Ca^2+^ homeostasis [12]. Protein folding is strongly related to the ER Ca^2+^ regulation. Disturbance in protein folding influences Ca^2+^ regulation, e.g. calnexin was shown to inhibit SERCA [30]. Calreticulin, which is a major Ca^2+^ storage protein [30] was also shown to be increased by malformed proteins [31]. On the other hand, ER Ca^2+^ depletion can influence the functionality of several chaperon proteins regulating ER-protein traffic [32]. Brostrom et al. argues that Ca^2+^ is playing a pivotal role in diverting resources from protein synthesis to energy production during stress situation to maintain ATP levels [33]. Thus depletion of ER Ca^2+^ during stress reduces protein synthesis, while triggers increased expression of ER resident chaperons like glucose-regulated protein (Grp78) which leads to UPR. In our present experiments, lithium induced increased levels of stored (ionomycin resistant) Ca2+ in Jurkat cells, although less prominently than in yeast models. We also found that basal intracellular free Ca2+ levels were elevated in starved cells but not in galactose or galactose/lithium treated cells. Since the rise of free Ca2+ is a crucial signal in apoptosis, maintaining relatively modest free Ca2+ could be a key factor for these cells to be more resistant to cell death.

Glucose starvation leads to ER stress and UPR [34]. Interestingly, galactose [12] as well as lithium [35] has both been separately reported to increase UPR. In the case of galactose, toxic metabolite levels were suggested as a direct cause of malfolded proteins and subsequent UPR. Lithium might have a much more beneficial role: by inducing the expression of chaperons like Grp78, Grp94 and calreticulin, it can be actually protective in ER stress caused by other factors [36]. In our present study, we found that XBP-1 and especially the splicing variant XBPS significantly elevated in Jurkat cells simultaneously exposed to both lithium and galactose. Interestingly, UPR activation was even more pronounced in galactose/lithium treated cells than in starving cells. Taken together with the fact that these cells possessed better survival and relatively moderate change in Ca^2+^ levels, this suggest that lithium might be protective against the relative hypoglycemia and the toxic downstream metabolite levels of galactose.

Hypoglycemia was already shown to decrease the number of N-glycans per protein [37] and to shorten the oligosaccharide chains to Man_2_GlcNAc_2_ or Man_5_GlcNAc_2_ [38]. Isono recently demonstrated [18] that the activation of nucleocytoplasmic O-Glycosylation (O-GlcNAc) in glucose starved cells is in reality the appearance of several GlcNAc_2_ modified N-glycan proteins due to the cross-reactivity of the anti-O-GlcNAc antibody CTD110.6. In contrast to N-type glycosylation, O-GlcNAc means the addition of O-linked N-acetylglucosamine to proteins on the Ser/Thr residues. It is a reversible process and occurs in both in cytoplasm and the nucleus [19]. In our present study, we found that galactose-grown, lithium treated Jurkat cells showed significantly elevated levels of CTD110.6 positivity, however this positivity almost entirely disappeared upon PNGase treatment which removes only N-linked N-acetylglucosamine from the protein backbone, but leaves O-GlcNAc intact. Thus, our results were similar to data published by Isono [18]. It has to be mentioned that all of our experiment was done only on Jurkat cells. Therefore we can not exclude the possibility that other cell types would behave differently, thus further studies need to be carried out to clarify whether the aforementioned processes are exclusive for Jurkat or they are more general features among various cell types. We also can not exclude that O-GlcNAc was also enhanced following galactose/lithium treatment parallel to N-linked GlcNAc_2_, but in our experimental setup, it appeared to be negligible. More importantly, CTD110.6 reactive proteins showed very similar Western-blot pattern in these cells, compared to starved cells. The dynamics of these modified proteins were slightly different though; the amount of GlcNAc_2_ carrying proteins quickly elevated then stabilized at a plateau phase in galactose/lithium treated cells, while in fasting cells GlcNAc_2_ seemed to be continuously increase until cells were dying.

Various alterations in N-glycosylation pattern has been described when galactose metabolism is disturbed, most notably in galactosemic patients [16], [39], however to our knowledge this is the first time to describe the appearance of GlcNAc_2_ moiety in relation to altered galactose metabolism. Clarifying the exact mechanism that induces the forming of these incomplete oligosaccharides will probably require studying the expressional level and functionality of a series of enzymes that are involved in dolichol-linked oligosaccharide biosynthesis. Isono argues that glucose deprivation might imitate some of the enzymatic phenotype observed in various types of carbohydrate-deficient glycoprotein (CDG) syndrome [18]. Sturiale et al. [16] suggest that galactosemia is also similar to CDG. It is also suggested that the defect(s) of the Leloir pathway in galactosemic patients cause elevated Gal-1P and decreased UDP-Gal that interferes with glycosyltransferases involved in oligosaccharide synthesis [40]. We assume that in our experimental setup; growing cells on galactose and blocking PGM by lithium might influence N-glycan synthesis by a mixture of relative glucose deprivation and altered galactose metabolite levels.

Experimental models of galactosemia are usually based on yeast cells. We think that influencing galactose metabolism by lithium in cell cultures might provide useful information about the molecular mechanisms involved in galactosemia. Here we showed that lithium induced changes in post-translational modification concerning oligosaccharide moieties that are reminiscent of galactosemia. Interestingly, it was suggested by Bhat that inositol depletion by the inhibition of inositol monophosphatase (IMPase) (one of the proposed mechanisms of lithium) might be also related to galactose metabolism [41]. In this hypothesis, lithium could cause an accumulation of Gal-1P which is a regulator of IMPase. Thus, bipolar disease and galactosemia could be considered as the two end-point of a delicate balance of Gal-1P.

Our results showing that altered N-glycan synthesis could occur when lithium influences galactose metabolism evoke some noteworthy implication in bipolar disease. Elevated serotonin transporter binding was shown to be associated with depressive disorders [42], it was also shown that proper N-glycan synthesis and protein folding is necessary for serotonin transporter assembly [43]. We think that lithium might have a beneficial effect by partially blocking N-glycan synthesis of these transporters through the influence on galactose metabolism.

Disturbances in N-glycan synthesis can also impact the outcome in AD since amyloid precursor protein is a membrane spanning glycoprotein. Surprisingly, increased level of bisecting GlcNAc residues on N-glycans - that were also shown in abundance on proteins in galactosemia [40] - was found to be protective in AD [44]. The other hallmark of AD, tau protein was shown to be glycosylated only in AD brains, but not in normal [45]. Although a recent clinical study found that lithium treatment decreased the prevalence of AD in bipolar patients [46], it requires further investigation to clarify whether it was mediated by its effect on N-glycan synthesis.

The immune system is also heavily dependent on proper N-glycan synthesis [39], [47], but to our knowledge, there is yet no available information about the impact of lithium on the immune system in this respect. Although lithium seems to modulate the immune system [48], its action is controversial. While autoimmune thyreoiditis is associated with bipolar disease, and it is probably exacerbated by chronic lithium treatment [49], another study showed that lithium prevents experimental autoimmune encephalomyelitis [50]. Since lithium's effect was mostly investigated by its impact on GSK-3 and related signaling pathways, we propose that analyzing N-Glycan profiles would contribute to the better understanding of its influence on the immune system.

In conclusion, we have shown that lithium treatment in galactose grown Jurkat cells significantly increased the level of proteins seemingly carrying O-GlcNAc but we have proven that these proteins rather contained N-linked GlcNAc moiety. This change of post-translational modification was coincided with the activation of UPR. Glucose deprived cells showed similar stress response, however galactose/lithium treated cells had significantly better survival rate. Therefore we suggest that inhibiting PGM by lithium is a viable option to study ER-related stress response pathways. These data, in combination with data published in our earlier reports [6], [11] suggest that disturbances in galactose metabolism strongly influence various processes that are associated with the ER, such as carbohydrate metabolism, Ca^2+^ regulation, or protein post-translational modification.

## Supporting Information

Figure S1 **Jurkat cells continue to grow in galactose containing media.** Equal amount of Jurkat cells were and grown in 5 mM glucose alone or 5 mM glucose +5 mM galactose containing medium. Without media replacement, the cells were left to grow for the indicated times (0, 1, 2, 3 or 4 days). (**A**) Each day the remaining glucose content of the media (▴) was measured. Plotted data points are combined means of both groups (±SD) from 3 independent experiments (There was no statistically significant difference between the glucose consumption rate of glucose-fed or glucose plus galactose-fed cells). Also, equal volume of cell suspensions were centrifuged and the cell pellet was lysed to extract proteins. Protein concentrations were measured to estimate the total cell mass for both conditions (♦ 5 mM glc alone, ◊ 5 mM glc +5 mM gal). (**B**) Left: Immunoblot analysis of the proteins extracts stained with CTD110.6 antibody. Right: Densitometric analysis of the total CTD110.6 staining over time (♦ 5 mM glc alone, ◊ 5 mM glc +5 mM gal). Levels are expressed as a percentage of the baseline intensity. Cells grown solely on glucose are producing specific type of N-gycans detectable by CTD110.6 after all the glucose in the media is consumed (from day 3), while at the same time cells grown on glucose plus galactose switch to metabolize galactose to avoid hypoglycemia thus CTD110.6 positivity remains relatively low.
(TIF)

Figure S2 **Densitometric analysis of CTD110.6 and WGA blots shown in** **Figure 7** **.** Densitometry of western-blots using CTD110.6 antibody and peroxidase-conjugated lectin (WGA) staining. Jurkat cells were previously incubated for 48 hours in 5 mM glucose, 5 mM galactose or hexose-free media, supplemented with either 0, 2.5 or 10 mM lithium. (**A**) Densitometric analysis of the total CTD110.6 staining. (**B**) Densitometry of the total WGA staining. (**C**) Densitometric analysis of the CTD110.6 positive band indicated in Figure 8A by the arrow. (**D**) Densitometric analysis of the WGA positive band indicated in Figure 8B by the arrow. Data are means ±SD from 3 independent experiments after normalized for total protein staining. *P<0.05 vs. 5 mM glc.
(TIF)
