# Supplementary figures and images for: Lithium Induces ER Stress and N-Glycan Modification in Galactose-Grown Jurkat Cells
Source: PLoS One. 2013 Jul 22;8(7):e70410. doi: 10.1371/journal.pone.0070410 (PMC3718757; doi:10.1371/journal.pone.0070410)

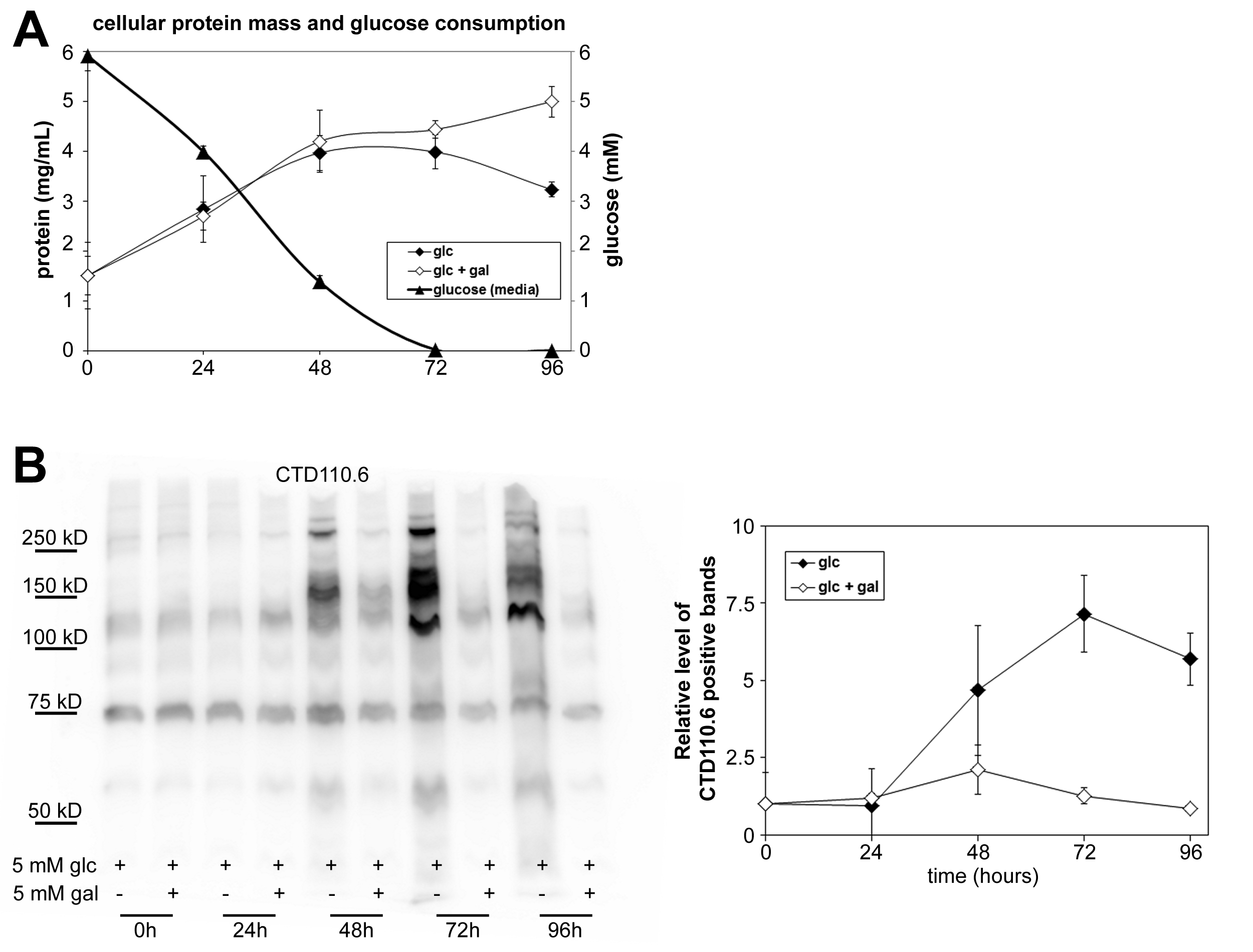

Supplement: Figure S1 — Jurkat cells continue to grow in galactose containing media. Equal amount of Jurkat cells were and grown in 5 mM glucose alone or 5 mM glucose +5 mM galactose containing medium. Without media replacement, the cells were left to grow for the indicated times (0, 1, 2, 3 or 4 days). (A) Each day the remaining glucose content of the media (▴) was measured. Plotted data points are combined means of both groups (±SD) from 3 independent experiments (There was no statistically significant difference between the glucose consumption rate of glucose-fed or glucose plus galactose-fed cells). Also, equal volume of cell suspensions were centrifuged and the cell pellet was lysed to extract proteins. Protein concentrations were measured to estimate the total cell mass for both conditions (♦ 5 mM glc alone, ◊ 5 mM glc +5 mM gal). (B) Left: Immunoblot analysis of the proteins extracts stained with CTD110.6 antibody. Right: Densitometric analysis of the total CTD110.6 staining over time (♦ 5 mM glc alone, ◊ 5 mM glc +5 mM gal). Levels are expressed as a percentage of the baseline intensity. Cells grown solely on glucose are producing specific type of N-gycans detectable by CTD110.6 after all the glucose in the media is consumed (from day 3), while at the same time cells grown on glucose plus galactose switch to metabolize galactose to avoid hypoglycemia thus CTD110.6 positivity remains relatively low. (TIF) [file pone.0070410.s001.tif]

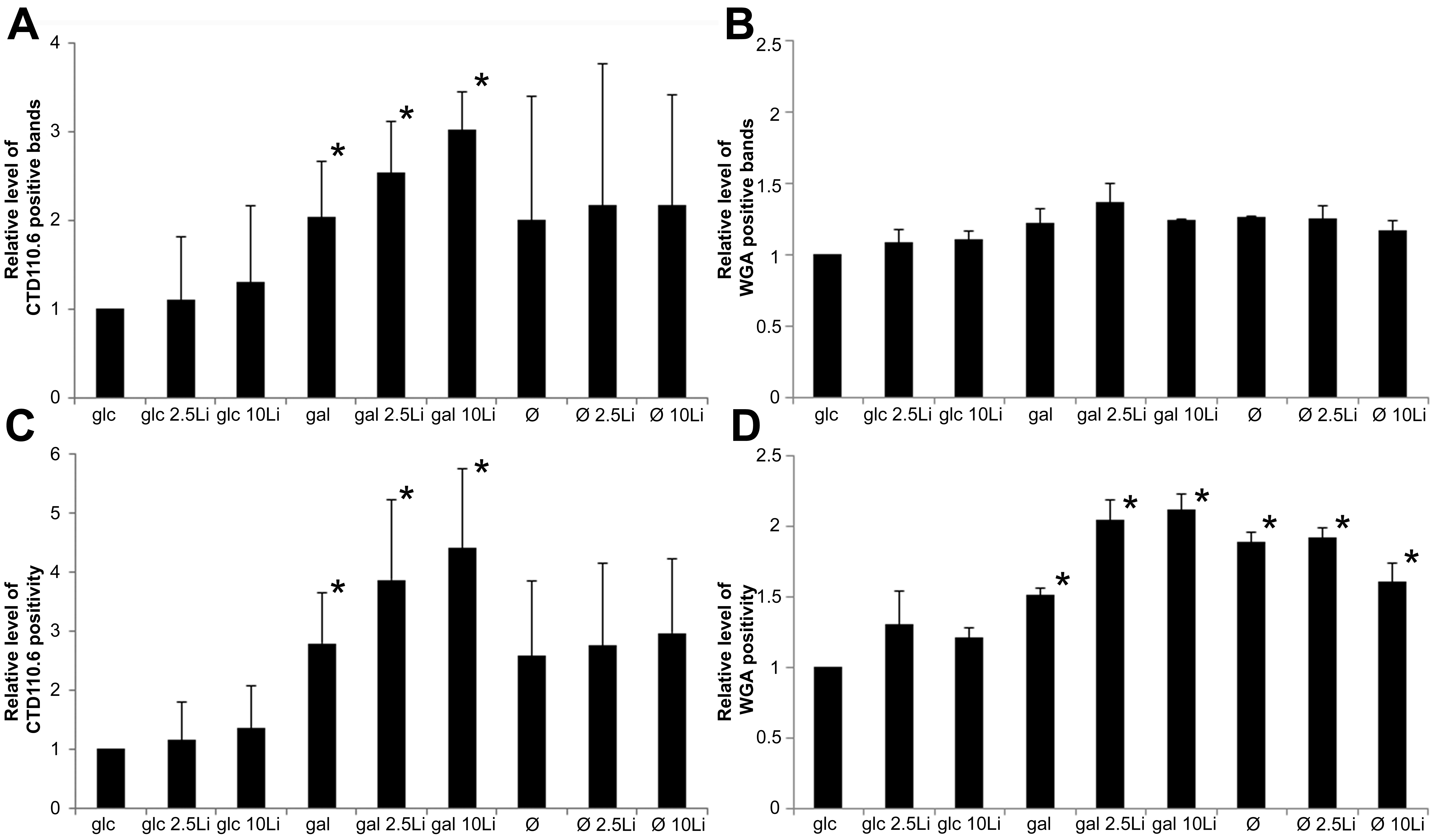

Supplement: Figure S2 — Densitometric analysis of CTD110.6 and WGA blots shown in Figure 7 . Densitometry of western-blots using CTD110.6 antibody and peroxidase-conjugated lectin (WGA) staining. Jurkat cells were previously incubated for 48 hours in 5 mM glucose, 5 mM galactose or hexose-free media, supplemented with either 0, 2.5 or 10 mM lithium. (A) Densitometric analysis of the total CTD110.6 staining. (B) Densitometry of the total WGA staining. (C) Densitometric analysis of the CTD110.6 positive band indicated in Figure 8A by the arrow. (D) Densitometric analysis of the WGA positive band indicated in Figure 8B by the arrow. Data are means ±SD from 3 independent experiments after normalized for total protein staining. *P<0.05 vs. 5 mM glc. (TIF) [file pone.0070410.s002.tif]
